# Normalized compression distance to measure cortico-muscular synchronization

**DOI:** 10.3389/fnins.2022.933391

**Published:** 2022-11-10

**Authors:** Annalisa Pascarella, Eugenia Gianni, Matteo Abbondanza, Karolina Armonaite, Francesca Pitolli, Massimo Bertoli, Teresa L’Abbate, Joy Grifoni, Domenico Vitulano, Vittoria Bruni, Livio Conti, Luca Paulon, Franca Tecchio

**Affiliations:** ^1^Institute for the Applications of Calculus “M. Picone”, National Research Council, Rome, Italy; ^2^Laboratory of Electrophysiology for Translational NeuroScience, Institute of Cognitive Sciences and Technologies, Consiglio Nazionale delle Ricerche, Rome, Italy; ^3^Unit of Neurology, Neurophysiology, Neurobiology, Department of Medicine, University Campus Bio-Medico of Rome, Rome, Italy; ^4^Department of Basic and Applied Sciences for Engineering (SBAI), University of Rome “La Sapienza”, Rome, Italy; ^5^Faculty of Psychology, Uninettuno University, Rome, Italy; ^6^Department of Neuroscience, Imaging and Clinical Sciences, University “Gabriele D’Annunzio” of Chieti-Pescara, Chieti, Italy; ^7^Faculty of Engineering, Uninettuno University, Rome, Italy; ^8^Istituto Nazionale di Fisica Nucleare, Sezione Roma Tor Vergata, Rome, Italy; ^9^Independent Researcher, Rome, Italy

**Keywords:** normalized compression distance (NCD), electrophysiology, handedness, neuronal synchronization, feedback

## Abstract

The neuronal functional connectivity is a complex and non-stationary phenomenon creating dynamic networks synchronization determining the brain states and needed to produce tasks. Here, as a measure that quantifies the synchronization between the neuronal electrical activity of two brain regions, we used the normalized compression distance (NCD), which is the length of the compressed file constituted by the concatenated two signals, normalized by the length of the two compressed files including each single signal. To test the NCD sensitivity to physiological properties, we used NCD to measure the cortico-muscular synchronization, a well-known mechanism to control movements, in 15 healthy volunteers during a weak handgrip. Independently of NCD compressor (Huffman or Lempel Ziv), we found out that the resulting measure is sensitive to the dominant-non dominant asymmetry when novelty management is required (*p* = 0.011; *p* = 0.007, respectively) and depends on the level of novelty when moving the non-dominant hand (*p* = 0.012; *p* = 0.024). Showing lower synchronization levels for less dexterous networks, NCD seems to be a measure able to enrich the estimate of functional two-node connectivity within the neuronal networks that control the body.

## Introduction

The neurons of the various brain areas communicate with each other through fluctuating signals in dynamic synchrony (Varela et al., 2001) both during rest and while performing tasks (Deco et al., 2011). By sustaining communication among networks (Fries, 2005), synchronization of neural activity mediates information processing in the brain (Singer, 1993; Borisyuk et al., 1998; Fries, 2009). In other words, the correlated neurons’ behaviors, even though they are generated by spatially discrete and/or distant areas (Gray et al., 1989), emerge from the integration of their signals that allow for sensory (Gray, 1994), attentional (Womelsdorf and Fries, 2007), or motor processing as well as for memory (Axmacher et al., 2006), and for other fundamental cognitive processes (Daffertshofer and Pietras, 2020). As a classical example, in visual areas, phase-locked oscillations of spatially segregated neuronal pools mediate binding of diverse visual features like motion, shape, and color into a coherent perception (Singer and Gray, 1995). Consistently, the multimodal neuroscience community converges in viewing the brain as a neuronal network where the nodes of the network represent either distinct cortical/subcortical areas, neuronal pools, or even single neurons and the edges represent their connections, that is their functional connectivity (FC) (Bullmore and Sporns, 2012; Wang et al., 2014). FC across distinct nodes is assessed as a statistical dependence among their signal times series measured through one of multiple methodologies from electro-encephalography (EEG) to magneto-encephalography (MEG) to functional-magnetic resonance imagining (fMRI) (Hutchison et al., 2013; Wang et al., 2014).

Functional connectivity among and within brain networks even in resting state clearly emerging by diverse technologies like fMRI (Damoiseaux et al., 2006) and EEG (Samogin et al., 2020) are characterized by specific frequency and spatial domains. In the case of motor behavior, FC emerges within and among several areas of the central nervous system—such as the motor, frontal, parietal, premotor cortices, subcortical, and cerebellar areas, as well as the spinal cord—finally expressing in the muscles’ contractions (van Wijk et al., 2012). Fine motor commands resulting from central processing (Lemon, 2008; Moreno-López et al., 2016) parallel the synchronization features of the electrical activity recorded on the surface of the muscles with those of primary sensorimotor cortex (Wolpert et al., 2011).

Linear measures (coherence and correlation) have been traditionally used to assess the degree of functional connectivity among different nodes (Gross et al., 2001; Broyd et al., 2009; Jensen and Mazaheri, 2010). Notably, some authors noticed that the absence of a linear statistical link between two nodes does not mean absence of FC (Fingelkurts et al., 2005). This is one of the reasons why non-linear measures of FC, such as mutual information, are attracting more and more attention (Hlinka et al., 2011; Wang et al., 2016).

In the case of cortico-muscular synchronization, a classically used electrophysiological measure is the cortico-muscular coherence (CMC) (Mima and Hallett, 1999; Liu et al., 2019). This is the spectral coherence between the EEG or MEG signal from the contralateral cortex and electro-myographic (EMG) signal recorded by involved muscles while executing a motor task. CMC showed how neurons synchronize their firing patterns at different frequencies according to diverse behavioral states (Mima and Hallett, 1999) as for example as a function of different force levels of contraction (Brown et al., 1998; Mima et al., 1999; Brown, 2000), initiating movement (Ramayya et al., 2021), exerting either a static force (Kristeva et al., 2007) or dynamic ones (Omlor et al., 2007).

Though CMC is considered a well-established index of cortex-muscle information flow both in healthy and pathological conditions (Mima and Hallett, 1999; Liu et al., 2019), clear limitations emerged (Yang et al., 2018). Recently, we measured CMC sensitivity to visual feedback information and handedness, while participants were performing a weak handgrip task with the right or the left hand with or without undirect visual feedback (L’Abbate et al., 2022). Tough we observed sensitivity of CMC to visual feedback, no significant variation of CMC related to handedness emerged, nor was it present in previous literature (Tecchio et al., 2006). Therefore, given the central role of asymmetries in the functioning of our body-brain system and the importance of handedness in our everyday lives, we hypothesized that limit in assessing such crucial feature originates from the CMC measure itself.

We propose here that measures sensitive to the complex nature of the exchanged signals can be sensitive to the differences in the organization of cortical areas controlling the two hands. Accordingly, other authors pointed out the limitations of linear electrophysiological measurements in view of the known features of the sensorimotor system (Yang et al., 2016, 2018; Tan et al., 2022). For example they observed that, while the synchronization in the sensorimotor system originates from ascending somatosensory feedback and descending motor commands (Kilner et al., 2004; Witham et al., 2011), CMC cannot separate this bidirectional contribution in cortico-muscular interaction. Moreover, they observed that, from the last studies the sensorimotor system appears to be non-linear, showing cross-frequency coupling (Chen et al., 2010; Yang et al., 2018), paving the way to non-linear measures able to complement linear ones (Palva et al., 2005; Yang et al., 2016; Siebenhühner et al., 2020).

Here we propose to study FC using a novel non-linear measure, the normalized compression distance (NCD). It is a parameter-free measure that estimates the information shared by two signals by comparing the compression length of a file obtained concatenating one signal with the other. NCD seems suitable for biological systems, as it yields excellent results in comparing genomes, clustering languages or music (Li and Vitányi, 1990). Notably, NCD is robust in the sense that its performance appears somewhat independent of the type of compressor used for coding the data. NCD does not require any features or background knowledge about the data. We selected this synchronization measure because it estimates the information shared by the two signals without requiring any representation of the individual signal in harmonics and does not require the signal to be stationary. In fact, the hypotheses of stationary signal and the representation with sinusoid functions condemn the estimates to be insensitive to relevant parts of the interior dynamics of the neuronal pool activity (Buzsáki, 2009; Buzsáki et al., 2013; Cottone et al., 2017; Armonaite et al., 2021).

### Study aim

The design of our study is to test the NCD sensitivity to fundamental physiological features.

Aware of the lack of a gold standard for the FC quantification we propose here a heuristic approach to find a solution that better reflects the state of the art of the interrelation among networks. In other words, we search for a FC measure sensitive to the networks’ ability, which is well known to depend on its FC levels. In fact, it is an established notion that neural networks in their resting state express characteristics relating to their ability to perform the functions in which they are involved (Deco and Corbetta, 2011; Kim and Kang, 2018; Bansal et al., 2021; Doucet et al., 2022). On this basis, we expect that there will be functional measures that differ between cortical representation of the dominant and non-dominant hand even when tested via a simple task. Accordingly, in our experience with the primary somatosensory area, the activation properties of networks with different levels of ability, particularly the thumb and little finger representation networks, differed when tested while responding to elemental galvanic stimulation (Tecchio et al., 2007). Based on this reasoning, we expect that even tested by a simple handgrip—performed with the same quality by the left hand and the right hand (L’Abbate et al., 2022)—we can perceive the differential organization of the representation networks of the two hands by NCD.

As paradigmatic example we studied with the NCD the synchronization between cortex and muscle (CMncd) while executing a simple movement typical of everyday activity. Higher CMncd corresponds to lower synchronization. Especially we pose the working hypothesis that CMncd will show dependence on hand executing the task and the level of visual feedback. That is, we expect that: (i) left non-dominant hand control will express higher CMncd than right dominant hand control and (ii) providing undirect visual feedback CMncd will increase, as suggested by the behavior of cortico-muscular coherence (L’Abbate et al., 2022). To test the two working hypotheses, we collected EEG and EMG simultaneously when subjects were performing a weak isometric handgrip task, with either the right or left hand, with or without undirect visual feedback of their exerted pressure.

## Materials and methods

### Study design

The study was approved by the Ethical Committee of S. Giovanni Calibita Hospital, Rome, Italy (Figure 3C). It was a cross-over investigation study with two interacting conditions (moved hand and visual feedback). Since our goal was to test CMncd sensitivity to diverse levels of network ability, in the present work we considered the representations of the dominant and non-dominant hand while executing a mono-lateral weak handgrip in presence or absence of undirect visual feedback.

### Participants

Fifteen healthy volunteers (10 females and 5 males, age range from 22 to 48 years with mean 29 ± 6 years) participated in the study after signing a written informed consent. All subjects were right-handed (as tested by Edinburgh Handedness Questionnaire Oldfield, 1971), and had normal or corrected-to normal vision.

### Experimental procedure

#### Behavioral scoring

The fine hand-motor control was evaluated with the 9-hole peg test (Wang et al., 2015) executed by the right and left hands.

#### Electro-encephalography, electro-myographic, electrooculography, and electrocardiogram data recordings

The individual EEG (Brain Products GmbH, Munich, Germany) was recorded using a 64-channel acti-CHamp System with montage according to the 10-10 EEG International System and referenced to the Fz electrode. Electrode impedances were maintained below 5 kΩ. Surface EMG—recorded by using Ag–AgCl cup electrodes—of the right and left opponents pollicis muscle (EMG_OPr_ and EMG_OPl_) were recorded with a belly tendon montage. EEG and EMG were sampled at 5 kHz (pre-sampling analogical band pass filtering 0.1–2,000 Hz) and stored for off-line processing.

#### Visuo-motor task

**FIGURE 1:**
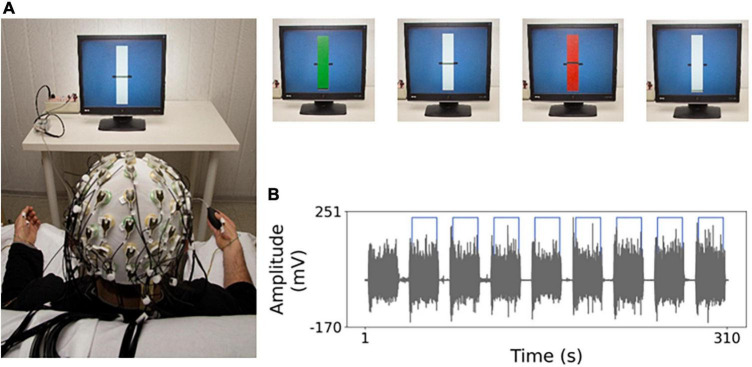
Experimental setting. **(A)** Electro-encephalography (EEG) recordings and task. The general set-up to record the EEG during the weak handgrip executed in sequences of 20 s starting with a go signal (green rectangle) and ending with a stop signal (red rectangle) intermingled by 10 s at rest. In the visual feedback “yes” (“no”) condition, a horizontal segment indicates the level of exerted pressure on the bulb by vertical oscillations (blocked). After determining handgrip maximum voluntary contraction (MVC), a rest period of at least 2 min was provided. Then, the weak isometric handgrip and rest sequences lasted about 5 min. The target level was set to 5% MVC, to minimize weariness related to the task. **(B)** Example of electro-myographic (EMG) acquisition during isometric contraction execution. In gray the EMG trace of opponents pollicis (OP) muscle in one representative subject, for the whole task duration, with 20 s contraction sequences intermingled by 10 s at rest. Light blue line indicates the temporal portions selected for analysis.

Each subject sat on a chair in front of a monitor at about 1 m (Figure 1A). As detailed in Figure 1 legend, the subject performed a handgrip, either with left or the right hand separately, against the resistance of a semi-compliant air-bulb, connected to a digital board that recorded the exerted pressure (Interactive Pressure Sensor, InPresS; Tomasevic et al., 2013). Notably, the visual information about the exerted pressure provided as a horizontal segment vertically oscillating on the monitor implies translated feedback, different from the physiological information that we usually have from our visual system while executing a movement, including a weak handgrip.

The four handgrips (about 5 min each) were executed in the same order in all subjects: first with the dominant hand with visual feedback (DxYes), then without (DxNo), thereafter with the non-dominant hand with (SnYes) and without visual feedback (SnNo).

### Data analysis

#### Electro-encephalography data pre-processing

Electro-encephalography data were filtered (1–250 Hz) before the analysis. A semi-automatic fast independent component analysis (fastICA)-based procedure (Barbati et al., 2004) was applied to the whole recordings to identify and remove biological (cardiac, ocular, and muscular) and non-biological (power line, instrumental, and environmental noise) artifacts. For each subject we selected about 180 s of artifact free signal for carrying the analysis. As preliminary step, we selected the bipolar derivations with maximal peak amplitude of cortico-muscular coherence in beta band in each condition (L’Abbate et al., 2022).

#### Normalized compression distance

The NCD is a quasi-universal metric, in the sense that it has been defined to simultaneously detect all similarities between signals that other effective distances detect separately (Cilibrasi and Vitányi, 2005). In other terms, NCD is based on the concept that two signals are similar if we can significantly “compress” one using the information of the other. NCD captures the dominant similarity over all possible features for every pair of signals compared, up to the stated precision.

We must remember that a lossless compressor acts as an invertible mapping function of a signal into a binary sequence. The length of this binary sequence reveals the amount of compression. Hence, the NCD computed between two signals x and y, i.e., NCD (x,y) is defined as


(1) $$\mathrm{NCD}\left(x,y\right)=\frac{C\left(\mathrm{xy}\right)\;-\;\min\left(C\left(x\right),C\left(y\right)\right)}{\max\left(C\left(x\right),C\left(y\right)\right)},$$

where C(xy) denotes the compressed size (length of the binary sequence that has been obtained by applying the compressor C) of the concatenation of x and y, wherein C(x) denotes the compressed size of x, and C(y) denotes the compressed size of y. NCD assumes values between 0 and 1, where 0 indicates maximum similarity and 1 the opposite.

In this work, the compressed size has been measured in terms of number of bits per sample, which is the average number of bits used for coding each sample of the considered signal. We used as compressor C the Huffman coding implemented in Matlab environment (CMncdH). To test the robustness of the proposed measure against the compressor, we computed the CMncd by using the Lempel–Ziv scheme (Lempel and Ziv, 1976) as compressor C. We used the normalized LZ proposed by Zhang et al. (2009) that takes into account the length of the sequence (CMncdLZ) (Zhang et al., 2009).

**FIGURE 2:**
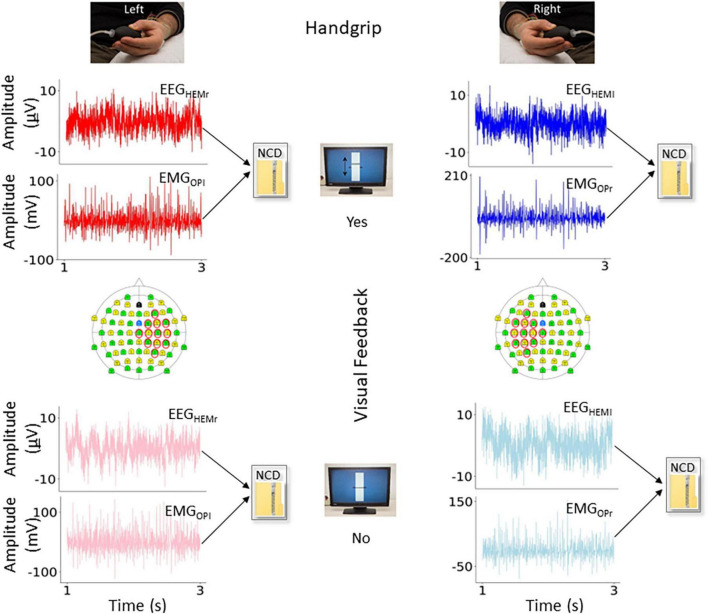
Variables of interest. In the four conditions of interest, representation of the functional connectivity measure obtained by normalized compression distance (NCD) from the electro-encephalography (EEG) and electro-myographic (EMG) ongoing signals. In red (left movement) and blue (right movement) the conditions with visual feedback (Yes), and in light red and light blue the conditions without (No). In the topographical representation of the 64-EEG recording channels, we highlighted those considered to estimate the bipolar derivation displaying highest cortico-muscular coherence (CMC) during the task with the contralateral hand, used as criterion to select the EEG representative.

For each subject and condition (Figure 2), we computed the CMncd between the cleaned EEG and EMG signals, with EEG being the selected bipolar channel, for epochs of 180 s length, windowed in segments of 18 s, obtaining 10 estimates for each subject and condition. In some subject we lacked the entire length and a minimum of 6–10 s intervals were included in all subjects.

### Statistical analysis

Preliminarily, we tested the stability of the CMncdH estimate by evaluating the variation coefficient of the about ten quantifications in successive 18 s epochs in the same condition, in all subjects and conditions.

The distribution of each variable was checked for normality by Shapiro–Wilk test and homogeneity of variance by Levene test. According to the variable distributions we applied the proper statistical analysis to identify CMncdH/CMncdLZ differences between dominant and non-dominant hand representations and presence and absence of undirect visual feedback. In other terms, once identified whether to apply parametric or non-parametric tests, we analyzed the comparisons across four conditions: *Hemi-Body* (left hemisphere-right hand, right hemisphere-left hand) and *Visual feedback* (Yes, No). We set the significance threshold at 0.05.

## Results

### CMncdH behavior across conditions

Shapiro–Wilk statistics indicated that the distribution of CMncdH and CMncdLZ measurements across subjects in the four conditions (DxNo, DxYes, SnNo, and SnYes) was not fitting a Gaussian. Furthermore, the Levene tests indicated that CMncdH and CMncdLZ displayed variances not homogeneous across the four conditions. On these bases, we applied non parametric statistical test searching for differences both between absence and presence of visual feedback (within the hemibody; e.g., when using the same hand) and between hemibody, e.g., between dominant and non-dominant hemibody within the same feedback condition (absence or presence).

### Huffman compressor

**FIGURE 3:**
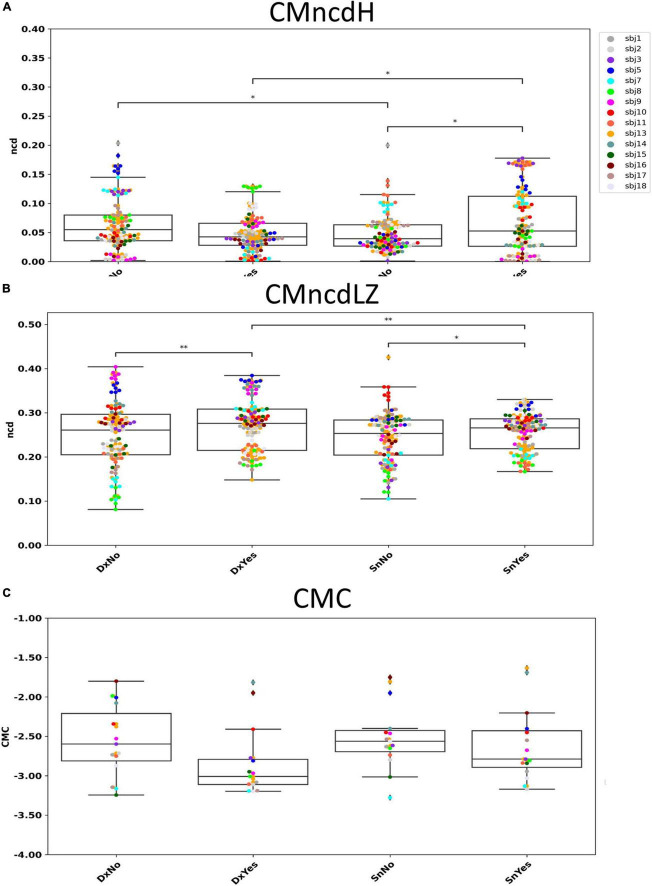
CMncdH and CMncdLZ dependence on behavioral condition. Boxplot of CMncdH **(A)** and CMncdLZ **(B)** reporting for each subject the value in 6 estimate intervals in the four conditions. Black lines with asterisks: conditions differing for *p* < 0.05 (1 asterisk) and *p* < 0.01 (2 asterisks). In panel **(C)** we show for comparison single subject data of the study L’Abbate et al. (2022), where we observed a that in absence of visual feedback cortico-muscular coherence (CMC) had higher amplitudes than in presence. Note that higher CMC corresponds to lower CMncd.

No significant difference was found by comparing the two conditions DxNo and DxYes across subjects (*W* = 1653; *p* = 0.112, Figure 3A). A significant difference was found by comparing the two conditions SnNo and SnYes across subjects (*W* = 1421; *p* = 0.012). Significant difference was found between the two conditions DxNo and SnNo across subjects (*W* = 1452; *p* = 0.016). A significant difference was found between the two conditions DxYes and SnYes (*W* = 1419; *p* = 0.011).

### Lempel–Ziv compressor

Significant difference was found by comparing the two conditions DxNo and DxYes across subjects (*W* = 1262; *p* = 0.0016, Figure 3B). A significant difference was found by comparing the two conditions SnNo and SnYes across subjects (*W* = 1488; *p* = 0.024). No significant difference was found between the two conditions DxNo and SnNo across subjects (*W* = 1883; *p* = 0.51). A significant difference was found between the two conditions DxYes and SnYes (*W* = 1378; *p* = 0.007).

### Behavioral quality of fine motor hand control

Execution times of the 9 Hole Peg Test displayed a distribution not differing from a Gaussian, and the ANOVA with *Hand* (right and left) and *Repetition* (1st and 2nd) as within-subject factors indicated a clear *Hand* effect [*F*_(1,15)_ = 8.42, *p* = 0.011] corresponding to quicker execution with the right (16.1 ± 0.4 s) than with the left hand (17.5 ± 0.3 s).

## Discussion

Our results show that both CMncdH and CMncdLZ (CMncd considering both) display higher values in absence with respect to presence of visual feedback when executing the left handgrip, reflecting minor synchronization between cortex and muscles when the task requires the integration of transposed visual feedback about the exerted pressure as per the working hypothesis. Moreover, in presence of visual feedback, CMncdH appears sensitive to laterality of movement: it displays higher values for the left than for the right handgrip. As expected, hand control of the non-dominant side expresses minor cortico-muscular synchronization than the dominant one.

Overall, the results tell us that CMncd is sensitive to motor control dexterity, differentiating the dominant vs. non-dominant sides for everyday movements, and revealing the difficulty of the non-dominant side to integrate unusual information during an unfamiliar task.

Similarly to CMncd, CMC (L’Abbate et al., 2022) showed sensitivity to visual feedback (Figure 3C). Indeed, we observed that the CMC peak in beta band appeared higher in amplitude in the everyday movement in absence of indirect visual feedback. The two measures CMncd and CMC consistently evidence that the unfamiliar task requiring integrating unusual information and focusing attention implies learning mechanisms reflected in a minor cortico-muscular synchronization, that is in a less tuned cortico-muscular communication.

On the other hand, CMncd during everyday movements revealed better synchronizations for the dominant than the non-dominant hand, not emerging in CMC. A possible explanation of higher sensitivity of CMncd is that synchronizations observed frequency by frequency miss synchronizations occurring through different signal patterns.

In planning and implementing motor actions, the gaze plays a crucial role: it both precedes and guides our everyday actions (Jovancevic-Misic and Hayhoe, 2009). When we perform an everyday action we implement eye-motor programmes in parallel with the execution of the spatial shifts of the body segments we are moving (Flanagan and Johansson, 2003). Definitely, it is acknowledged that visual feedback has an essential role for the motor control of hand movements (Saunders and Knill, 2004). The evidence shows that visual feedback is relevant for the on-line control of reaching movements (Saunders and Knill, 2003), grasping movements (Connolly and Goodale, 1999) and object manipulation (Johansson et al., 2001). In the simple handgrip movement exploited in our experiment, the typical physiological condition of everyday life is implemented in the “No” condition. In fact, although people did not look at their hand, the handgrip task looking at the fixed monitor reflects the typical condition in which at the table we eat by looking at each other and without continuously looking at our cutlery. In this situation, the cortico-muscular synchronizations did not differ when moving the dominant or the non-dominant hand.

The task where we provided via the position of the vertically fluctuating horizontal segment information about the executed pressure, is a motor execution quite different from our use of a whatever light tool, for which we calibrate the strength depending on visual and proprioceptive information (Sober and Sabes, 2005). In other terms, our “Yes” condition requires rapid adaptability involving learning mechanisms. In our findings, while the dominant side expressed similar features while executing the everyday movement or the unusual one, the non-dominant hemi-body expressed less cortico-muscular synchronization in approaching the management of novelty. Possibly, CMncd senses the difficulty that less dexterous system encounters to exploit the indirect transposed information with respect the manipulated object.

In our working hypothesis, derived from resting state knowledge, effects of handedness was expected to appear independently of the behavioral test. On the contrary, the effect emerged for movements not belonging to the everyday repertoire, when the two dominant and non-dominant controlling networks were involved in a task with unfamiliar processing requirements. This result leads to reason that while in central networks the resting state emerges with a continuous ongoing neuronal pools activity, the muscles are electrically silent at rest, so that the cortico-muscular synchronization is to be expected much more behaviorally dependent than the intra-cerebral networks’ activities.

The human brain, as well as other biological systems, presents asymmetries in structure and function (Toga and Thompson, 2003). It is suggested that lateralization emerged as a function of evolutionary, developmental, hereditary, and experiential factors (Corballis, 2003). Cerebral counterpart of lateralization of motor control was found in relation to skilled actions by EEG (Serrien et al., 2012; Serrien and Sovijärvi-Spapé, 2016) and brain imaging studies (Schluter et al., 2001). Our data strengthen the notion of diverse functional organizations of hemi-body homologue networks devoted to hand control. Indeed, when the subject is due to do a task with a relevant novelty processing component–as it can be adjusting the handgrip pressure according to a visual information distant and independent of the manipulated object–the network controlling the non-dominant left hand shows signs of less tuned coordination with respect to the dominant homolog.

Notably, CMncdH evidenced the dependence on hemi-body dominance also in everyday activities (DxNo vs. SnNo) and CMncdLZ evidenced the dependence on visual feedback also when moving the dominant hand. Further investigations are required to deep understanding of diverse compressors in sensing the physiological properties via NCD.

Tested on the motor-associated synchronization between cortical neuronal activity with that of muscular-sensed spinal moto-neurons, we introduce here the NCD as a measure of synchronization to consider the complex nature of the ongoing neuronal electrical activity, the neurodynamics. In the tested conditions, NCD sensitivity suggests that it can enrich the assessment of communication phenomena inside the nervous system, providing a new window to assess network functional connectivity properties. Because of its definition, NCD can be calculated between activities of different areas, even if collected at different times (Sarasa et al., 2019). This could be very useful when comparing the activities of a specific area at successive times along the lifespan or as an effect of a disease in longitudinal studies. NCD is also suitable for the comparison of signals with different lengths, for example, in the case of activities where artifacts occur in different periods and lead to incongruent epoch rejections (Li and Vitányi, 2008).

In conclusion, we believe that NCD can represent a relevant enrichment tool to assess synchronization phenomena between two nodes, thus enhancing the estimation of functional connectivity within the brain networks that support brain processing.

## Data availability statement

The raw data supporting the conclusions of this article will be made available by the authors, without undue reservation.

## Ethics statement

The studies involving human participants were reviewed and approved by the Ethical Committee of San Giovanni Calibita Hospital Fatebenefratelli, Rome, Italy. The patients/participants provided their written informed consent to participate in this study.

## Author contributions

FT and EG drafted the manuscript. AP, DV, and VB grounded the normalized compression distance theory and algorithm. MA and FP coordinated the analysis on the present data set. LC cured the manuscript writing. MB and JG deepened the clinical perspective and LP the conceptual framework. KA and TL’A shared the neurophysiology-psychotherapy connection. All authors contributed to the final writing.
